# A novel lncRNA-focus expression signature for survival prediction in endometrial carcinoma

**DOI:** 10.1186/s12885-017-3983-0

**Published:** 2018-01-05

**Authors:** Meng Zhou, Zhaoyue Zhang, Hengqiang Zhao, Siqi Bao, Jie Sun

**Affiliations:** College of Bioinformatics Science and Technology, Harbin Medical University, Harbin, 150081 People’s Republic of China

**Keywords:** Endometrial cancer, Long non-coding RNAs, Survival, Signature

## Abstract

**Background:**

Endometrial cancer (UCEC) is a complex malignant tumor characterized by both genetic level and clinical trial. Patients with UCEC exhibit the similar clinical features, however, they have distinct outcomes due to molecular heterogeneity. The aim of this study was to access the prognostic value of long non-coding RNAs (lncRNAs) in UCEC patients and to identify potential lncRNA signature for predicting patients’ survival and improving patient-tailored treatment.

**Methods:**

We performed a comprehensive genome-wide analysis of lncRNA expression profiles and clinical data in a large cohort of 301 UCEC patients. UCEC patients were randomly divided into the discovery cohort (*n* = 150) and validation cohort (*n* = 151). A novel lncRNA-focus expression signature was identified in the discovery cohort, and independently accessed in the validation cohort. Additionally, the lncRNA signature was evaluated by multivariable Cox regression and stratification analysis as well as functional enrichment analysis.

**Results:**

We detected a novel lncRNA-focus expression signature (LFES) consisting of 11 lncRNAs that were associated with survival based on risk scoring strategy in UCEC. The risk score based on the LFES was able to separate patients of discovery cohort into high-risk and low-risk groups with significantly different overall survival and progression-free survival, and has been successfully confirmed in the validation cohort. Furthermore, the LFES is an independent prognostic predictor of survival and demonstrates superior prognostic performance compared with the clinical covariates for predicting 5-year survival (AUC = 0.887). Functional analysis has linked the expression of prognostic lncRNAs to well-known tumor suppressor or ontogenetic pathways in endometrial carcinogenesis.

**Conclusions:**

Our study revealed a novel 11-lncRNA signature to predict survival of UCEC patient. This lncRNA signature may be a valuable and alternative marker for risk evaluation to aid patient-tailored treatment and improve the outcome of patients with UCEC.

**Electronic supplementary material:**

The online version of this article (10.1186/s12885-017-3983-0) contains supplementary material, which is available to authorized users.

## Background

Endometrial cancer, referred to as uterine corpus endometrial carcinoma (UCEC), is one of the most common gynecologic malignancy in the world with an increasing trend in recent years [1]. Surgical treatment is the primary treatment for UCEC patients. Although the 5-year survival rate for early diagnosed UCEC patients is around 80% [2], the prognosis of patients with advanced-stage or high risk of recurrence is poor [3]. Adjuvant therapy (radiation therapy and/or chemotherapy) after surgical treatment is associated with improved overall survival in high-risk patients [4]. However, adjuvant therapy may cause side effects that adversely impact patient’s quality of life. Therefore, it is urgent to develop prognostic or predictive biomarkers for risk evaluation to distinguish high- or low-risk patients and consequently make patient-tailored therapy.

Long non-coding RNAs (lncRNAs) were commonly defined as non-coding RNA molecules (ncRNAs) longer than 200 nucleotides (nt) in length distinguished from short ncRNAs [5]. Increasing evidence showed that lncRNAs is a key layer of genome regulatory network and play important roles in various fundamental biological processes through several main mechanisms such as signaling, decoying, scaffolding and guidance [6, 7]. Dysregulated expression of lncRNAs has widely been reported in various cancers and was recognized as a hallmark feature in cancer [8–10]. Recent studies have highlighted the clinical implications of lncRNAs as potential prognostic/diagnostic biomarkers or therapeutic targets in multiple cancers [11, 12]. Only several cancer-associated lncRNAs such as *MEG3*, *GAS5* and *SRA* were identified in UCEC [13–15]. To our knowledge, there are no prior studies of lncRNA expression profiles at a genome-wide scale focusing on the prognostic value of lncRNAs for survival prediction in UCEC.

In this study, we performed genome-wide analysis of lncRNA expression profiles integrating clinical data of 301 UCEC patients from The Cancer Genome Atlas (TCGA), and investigated the prognostic value of lncRNAs to identify a novel lncRNA-focus expression signature acting as a prognostic predictor for UCEC patients.

## Methods

### Patient datasets

Clinical and pathological characteristics of patients with UCEC tumors were retrieved from a previous study published by TCGA on May 01, 2013 [16]. In our study, we used a total of 301 patient samples with UCEC, which possessed paired lncRNA and mRNA expression profiles, survival information and classic clinicopathological factors. A brief summary of clinical factors of all samples was displayed in Table 1. All of UCEC patients used in this study were randomly divided into two patient cohorts for the purpose of discovery and validation, which results in a 150-sample discovery cohort and a 151-sample validation cohort. The details of clinical and pathological characteristics for both patient cohorts were listed in Table 1.

**Table 1:** Clinicopathological characteristics of UCEC patients used in this study

Variables		TCGA cohort (*n* = 301)	Discovery cohort (*n* = 150)	Validation cohort (*n* = 151)	*P*-value
Stage, no(%)	I	207 (68.8)	106 (70.7)	101 (66.9)	0.726^a^
	II	16 (5.3)	9 (6)	7 (4.6)	
	III	64 (21.3)	30 (20)	34 (22.5)	
	IV	13 (4.3)	5 (3.3)	8 (5.3)	
Grade, no(%)	1	70 (23.3)	33 (22)	37 (24.5)	0.619^a^
	2	81 (26.9)	38 (25.3)	43 (28.5)	
	3	150 (49.8)	79 (52.7)	71 (47)	
histology, no(%)	Endometrioid	243 (80.7)	124 (82.7)	119 (78.8)	0.664^a^
	Serous	50 (16.6)	22 (14.7)	28 (18.5)	
	Mixed	8 (2.7)	4 (2.7)	4 (2.6)	
Vital status, no(%)	Alive	270 (89.7)	133 (88.7)	137 (90.7)	0.69^a^
	Dead	31 (10.3)	17 (11.3)	14 (9.3)	
Age, years (mean ± SD)		63.4 ± 10.7	63.7 ± 11.1	63.0 ± 10.4	0.537^b^

^a^Chi square test

^b^Student’s t-test

### Acquisition and processing of mRNA and lncRNA expression profiles in UCEC patients

Genome-wide mRNA and lncRNA expression profiles (RPKM expression levels) were downloaded from TCGA long non-coding RNAs database (http://larssonlab.org/tcga-lncrnas/index.php) according to Akrami’s study [17]. Briefly, the acquisition and processing of mRNA and lncRNA expression profiles were performed by Akrami et al. as follows [17]: TCGA RNA-seq data in FASTQ format was realigned to the Hg19 assembly using TopHat software and read counts for each lncRNA and mRNA were obtained using HTSeq-count. Then, RPKM values were used to quantify expression levels of lncRNAs and mRNAs by normalizing for lncRNA or mRNA length and library size and were log transformed using log2 (RPKM + 0.01) [17]. A total of 20,462 mRNAs and 10,419 lncRNAs were finally retained in the further analysis.

### Statistical analysis

Univariate Cox regression analysis was used to select candidate prognostic lncRNAs that were significantly correlated with overall survival at the significance level of 1%. All candidate prognostic lncRNAs were subjected to the multivariate analysis with Cox proportional hazard model for identifying lncRNA biomarkers with independent prognostic value. The survival rate and median survival for each prognostic risk group were calculated using the Kaplan-Meier method. The survival difference between the high-risk group and the low-risk group was assessed by log-rank test with 5% significant level. Univariate Cox analysis was performed to evaluate the prognostic value of lncRNA signature. To assess the independence between lncRNA signature and the key clinical factors, multivariate Cox regression and stratification analyses were conducted. Hazard ratios (HRs) and 95% confidence intervals (CIs) were computed by the Cox analysis. The comparison of survival prediction based on lncRNA signature and key clinical characteristics were performed by the time-dependent receiver operating characteristic (ROC) analysis. Kruskal-Wallis test was used to compare expression levels for each lncRNAs across four UCEC subtypes. All statistical analyses were performed using R/Bioconductor.

### Formulation of lncRNA-focus expression signature

A multivariate Cox analysis was carried out by expression levels of these independent lncRNA biomarkers. Using the linear combination of lncRNA expression values weighted by the coefficients from the multivariate Cox analysis, the independent lncRNA biomarkers were integrated into a lncRNA-focus expression signature (LFES) by risk scoring method as shown in the following equations

$$ \mathrm{Risk}\  \mathrm{Score}\left(\mathrm{patient}\right)=\sum \limits_{\mathrm{i}=1}^{\mathrm{n}}\mathrm{coefficient}\left({\mathrm{lncRNA}}_{\mathrm{i}}\right)\ast \mathrm{expression}\left({\mathrm{lncRNA}}_{\mathrm{i}}\right) $$

Here, Risk Score(patient) is a LFES-based risk score for UCEC patient. lncRNA_i_ represents the ith prognostic lncRNA and expression(lncRNA_i_) is the expression level of lncRNA_i_ for the patient. Regression coefficient of multivariate Cox analysis was denoted as coefficient(lncRNA_i_) which represents the contribution of lncRNA_i_ for prognostic risk scores. Patients with higher risk score tend to have a poor survival outcome. The median risk score for discovery cohort was selected as the cutoff point. Based on this cutoff, patients in the discovery cohort, validation cohort and entire TCGA cohort can be assigned to a high-risk group or a low-risk group.

### In silico analysis of lncRNA function

Co-expression relationship was evaluated between lncRNAs and mRNAs using paired expression profiles of lncRNAs and mRNAs in entire TCGA UCEC patients, and lncRNA-mRNA co-expression network was constructed. Functional enrichment analysis of mRNAs in the lncRNA-mRNA co-expression network was used to infer potential biological processes and pathways of prognostic lncRNAs according to Gene Ontology (GO) and Kyoto Encyclopedia of Genes and Genomes (KEGG) through DAVID Bioinformatics Resources (https://david.ncifcrf.gov/, version 6.8) [18]. Finally, the top one of significantly enriched GO terms or KEGG pathways was considered as a potential function of prognostic lncRNAs.

## Result

### Patient’s characteristics

A total of 150 UCEC samples were randomly selected from 301 UCEC samples as discovery cohort, and other 151 UCEC samples composed the validation cohort. The details of clinical characteristics for both cohorts were listed in Table 1. The clinical variables, including stage, grade, histology and vital status, were similar in the training and validation cohorts. Results of the statistical analysis exhibited that the random assignment with the discovery and validation cohorts was in equilibrium with these clinical characteristics.

### Development of lncRNA-focus expression signature for survival prediction in UCEC

To identify prognostic lncRNAs distinguished between good survival and poor survival in UCEC patients, univariate Cox proportional hazards regression analysis for each lncRNA was carried out using the expression level in the discovery cohort. The initial 19 lncRNAs were identified to be significantly associated with survival with *p*-value <0.01 (Additional file 1). On the basis of the coefficients from univariate Cox regression, the lncRNA with negative coefficient was viewed as protective lncRNA. We found that the up-regulation of protective lncRNA was correlated with good overall survival. Oppositely, risky lncRNA with positive coefficient was associated with poor survival. In order to consider mutual effect among 19 lncRNAs, a multivariate analysis was performed to select optimal independent lncRNAs for survival prediction with the expression level of 19 candidate lncRNAs as covariates and overall survival as a dependent variable. We found that 11 out of 19 candidate lncRNAs with the significant *p*-value <0.1 were retained as the independent prognostic lncRNAs in UCEC. The list of 11 prognostic lncRNAs was shown in Table 2. Of these, only lncRNA *NRAV* was protective lncRNA with negative coefficient in univariate Cox analysis. All of the other 10 lncRNAs were risky lncRNA with positive coefficients.

**Table 2:** Univariate Cox regression analyses of the 11 lncRNAs associated with overall survival in UCEC

Ensembl id	Gene symbol	Genomic location	Coefficient	Hazard ratio	95% CI	*P* Value
ENSG00000260684	RP11-1072A3.3.1	chr16: 30,995,950–30,999,591	2.695	14.805	3.546–61.82	<0.001
ENSG00000229589	ACVR2B-AS1	chr 3: 38,451,027–38,454,820	1.038	2.823	1.522–5.237	0.001
ENSG00000224037	RP4-781 K5.7.1	chr1: 234,845,004–234,855,723	2.331	10.289	2.419–43.76	0.002
ENSG00000235499	AC073046.25	chr 2: 73,985,132–73,986,343	0.798	2.220	1.337–3.687	0.002
ENSG00000224905	AP001347.6	chr 21: 14,027,421–14,144,468	0.722	2.058	1.297–3.264	0.002
ENSG00000260992	DOCK9-AS2	chr 13: 99,087,819–99,088,625	0.306	1.358	1.11–1.661	0.003
ENSG00000248008	NRAV	chr 12: 120,490,328–120,495,940	−0.236	0.790	0.67–0.9313	0.005
ENSG00000234945	GTF3C2-AS1	chr 2: 27,335,535–27,342,599	4.013	55.321	3.258–939.3	0.005
ENSG00000182648	LINC01006	chr 7: 156,472,196–156,640,654	0.665	1.945	1.208–3.133	0.006
ENSG00000253636	RP11-531A24.5	chr 8: 73,052,178–73,063,061	0.410	1.507	1.107–2.052	0.009
ENSG00000233760	AC004947.2	chr 7: 26,551,822–26,557,200	0.172	1.187	1.042–1.353	0.010

To build a lncRNA-focus expression signature for survival prediction, lncRNA expression profiles of the selected 11 independent prognostic lncRNAs were used to build the multivariable Cox regression model for evaluating their relatively predictive power. We constructed lncRNA-focus expression signature (LFES) for survival prediction by weighted scoring method using expression level of independent prognostic lncRNAs weighted by their regression coefficients in above multivariate Cox analysis as follows: *Risk Score* (patient) = (5.0432 * expression value of *RP11-1072A3.3.1*) + (0.8462 * expression value of *ACVR2B-AS1*) + (6.3725 * expression value of *RP4-781 K5.7.1*) + (1.9110 * expression value of *AC073046.25*) + (1.9166 * expression value of *AP001347.6*) + (0.3553 * expression value of *DOCK9-AS2*) + (−0.2987 * expression value of *NRAV*) + (−6.896 * expression value of *GTF3C2-AS1*) + (−0.8517*expression value of *LINC01006*) + (0.5747 * expression value of *RP11-531A24.5*) + (0.2325 * expression value of *AC004947.2*).

### Prognostic validation of LFES in the discovery cohort

**Fig. 1:**
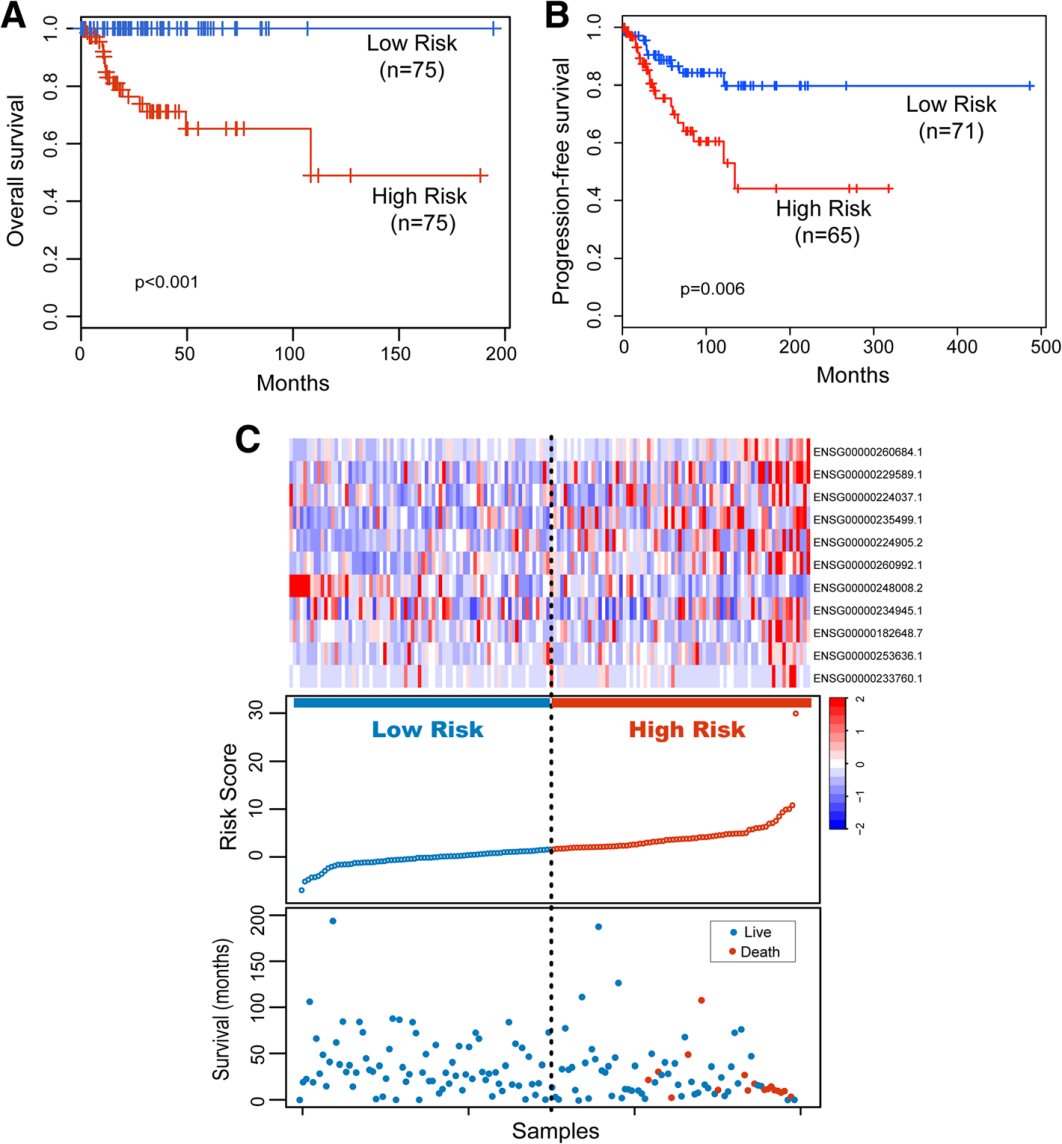
Prognostic assessment of the lncRNA signature in the discovery cohort. **a** Kaplan-Meier analysis for overall survival of patients in the predicted risk groups by the 11-lncRNA signature in the discovery cohort. **b** Kaplan-Meier analysis for progression-free survival of patients in the predicted risk groups by the 11-lncRNA signature in the discovery cohort. **c** Presentation of risk scores, survival status and lncRNA expression pattern in the predicted risk groups by the 11-lncRNA signature in the discovery cohort

To assess the prognostic value of the predictive model, a LFES-based risk score was generated for each patient in the discovery cohort by the expression level of 11 lncRNAs. The median risk score was obtained from the discovery cohort and was selected as the threshold point (1.703). According to the risk score and the threshold point, patients of discovery cohort were classified into high-risk group (*n* = 75) and low-risk group (*n* = 75). Survival analysis showed that there was a significant difference in overall survival (*p* < 0.001, log-rank test) (Fig. 1a) and progression-free survival (*p* = 0.006, log-rank test) (Fig. 1b) between patients in the high-risk group and low-risk group. As shown in Fig. 1a, patients in the high-risk group only have 3- and 5-year survival rates of 71.2% and 65.2%, respectively, compared to the patients in the low-risk group with 3- and 5-year survival rates of 100%. In a univariate Cox regression analysis, the hazard ratios of high-risk group versus low-risk for overall survival was 2.718 (*p* < 0.001, 95% confidence interval (CI) = 1.923–3.842) (Table 3).

**Table 3:** Univariate and Multivariate Cox regression analysis of the lncRNA signature and survival in different patient cohorts

Variables	Unfavorable/Favorable	Univariate			Multivariate		
		HR	95% CI	*P* vaule	HR	95% CI	*P* vaule
Discovery cohort (*n* = 150)							
11-lncRNA risk score	High/Low	2.718	1.923–3.842	<0.001	2.649	1.788–3.923	<0.001
Age		1.055	1.004–1.108	0.035	0.992	0.931–1.057	0.797
Stage	(III + IV)/(I + II)	4.122	1.588–10.7	0.004	1.219	0.392–3.795	0.732
Grade	3/(1 + 2)	4.397	1.258–15.37	0.020	1.609	0.397–6.52	0.506
Histology	Serous/Endometrioid	1.731	0.604–4.962	0.307	0.728	0.217–2.445	0.607
Validation cohort (*n* = 151)							
11-lncRNA risk score	High/Low	6.903	1.521–31.340	0.012	6.158	1.205–31.465	0.029
Age		1.042	0.986–1.101	0.141	1.046	0.975–1.122	0.208
Stage	(III + IV)/(I + II)	7.160	2.196–23.340	0.001	7.153	1.601–31.955	0.010
Grade	3/(1 + 2)	2.632	0.879–7.885	0.084	0.681	0.150–3.083	0.618
Histology	Serous/Endometrioid	4.873	1.627–14.600	0.005	0.691	0.099–4.830	0.709
Entire TCGA cohort (*n* = 301)							
11-lncRNA risk score	High/Low	11.767	3.568–38.810	<0.001	10.793	3.084–37.777	<0.001
Age		1.050	1.012–1.09	0.009	1.064	1.018–1.112	0.006
Stage	(III + IV)/(I + II)	4.835	2.359–9.906	<0.001	3.948	1.759–8.859	0.001
Grade	3/(1 + 2)	3.206	1.433–7.177	0.005	1.263	0.490–3.257	0.628
Histology	Serous/Endometrioid	2.584	1.236–5.402	0.012	0.509	0.209–1.240	0.137

The expression pattern of 11 prognostic lncRNAs, the distribution of the risk score and the survival status of UCEC patients for the discovery cohort was shown in Fig. 1c. Ten risky lncRNAs are over-expressed among patients with the high-risk score, but the protective lncRNA, *NRAV*, often would express in the low-risk cases.

### Further confirmation of LFES for survival prediction in the validation cohort and entire TCGA cohort

**Fig. 2:**
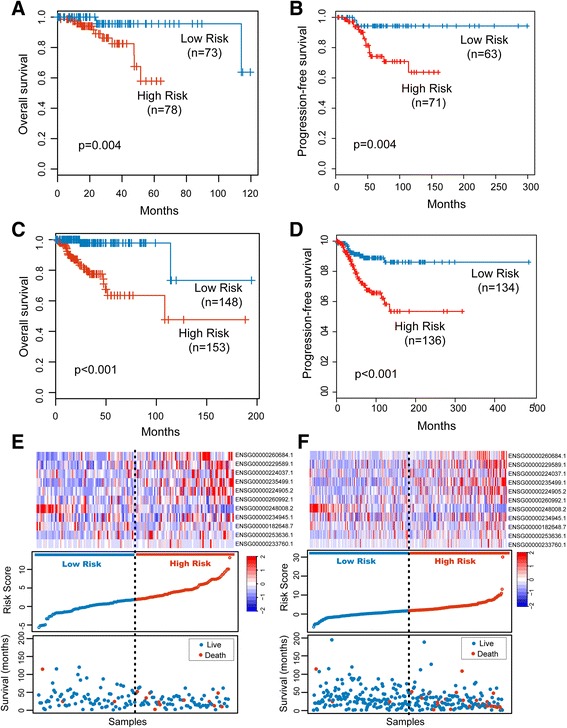
Independent validation of the lncRNA signature. Kaplan-Meier curves for overall survival of patients classified into high- and low-risk groups using the lncRNA signature in the validation cohort (**a**) and in the entire TCGA cohort (**c**). Kaplan-Meier curves for progression-free survival of patients classified into high- and low-risk groups using the lncRNA signature in the validation cohort (**b**) and in the entire TCGA cohort (**d**). The distribution of risk score, patients’ survival status and lncRNA expression pattern for high-risk and low-risk patients in the validation cohort (**e**) and in the entire TCGA cohort (**f**)

To validate the universality of LFES for identification of UCEC patients with poor outcome, we examined the ability of LFES in the independent validation cohort. By using the same LFES-based risk score model, the patients of the validation cohort were divided into high-risk group (*n* = 78) and low-risk group (*n* = 73) according to the same threshold point as for the discovery cohort. Patients with high-risk LFES had significantly shorter overall survival and progression-free survival than those with the low-risk signature (*p* = 0.004, log-rank test) (Fig. 2a and b). The 3- and 5-year survival rates of the high-risk group were 82.5% and 57.9%, respectively, whereas the corresponding rates in the low-risk group both were 95.6%. Notably, there were 11 cancer-related deaths in the high-risk group and only three death events in patients with low-risk scores. The hazard ratios of high-risk group versus low-risk group for overall survival was 6.903 (*p* = 0.012, 95% CI = 1.521–31.340) (Table 3).

We also elevated the prognostic value of LFES in the entire TCGA cohort. The LFES could also distinguish between patients with the good and poor outcome, which is consistent with the findings from the discovery and validation cohorts. Kaplan-Meier survival curves based on the LFES were significantly different (*p* < 0.001, log-rank test) (Fig. 2c and d). The median survival time for patients with high-risk scores was 108 months. In sharp contrast, the patients with low-risk scores had not reached the threshold to calculate their median survival time. The survival rates at 3- and 5-year were 77.5% and 63.5% for patients in the high-risk group compared with both 97.8% for patients in the low-risk group. By subjecting the risk scores to univariate Cox regression analysis, patients with high-risk scores exhibited an 11.767-fold increased risk than patients with low-risk scores (Table 3). The expression pattern of 11 prognostic lncRNAs, the distribution of the risk score and the survival status of UCEC patients for the validation and entire TCGA cohorts was shown in Fig. 2e and f, which is consistent with findings in the discovery cohort.

### Correlation between LFES and other clinicopathologic characteristics or subtype

To evaluate independent prognostic values of the LFES in survival prediction, we performed multivariate Cox regression analysis to test the performance of the LFES, including LFES-based risk scores, age, stage, grade and histology as covariates and overall survival as the dependent variable. In the discovery cohort, only the LFES was significant in multivariate analysis (*p* < 0.001, Table 3) compared to these clinical characteristics of age, stage and grade. Furthermore, the hazard ratios of high-risk group versus low-risk group for overall survival were 6.158 (*p* = 0.029, 95% CI = 1.205–31.465) in the validation cohort and 10.793 (*p* < 0.001, 95% CI = 3.084–37.777) in the entire TCGA cohort after adjustment by these clinical characteristics (Table 3), respectively, indicating that the LFES maintained an independent correlation with overall survival.

**Fig. 3:**
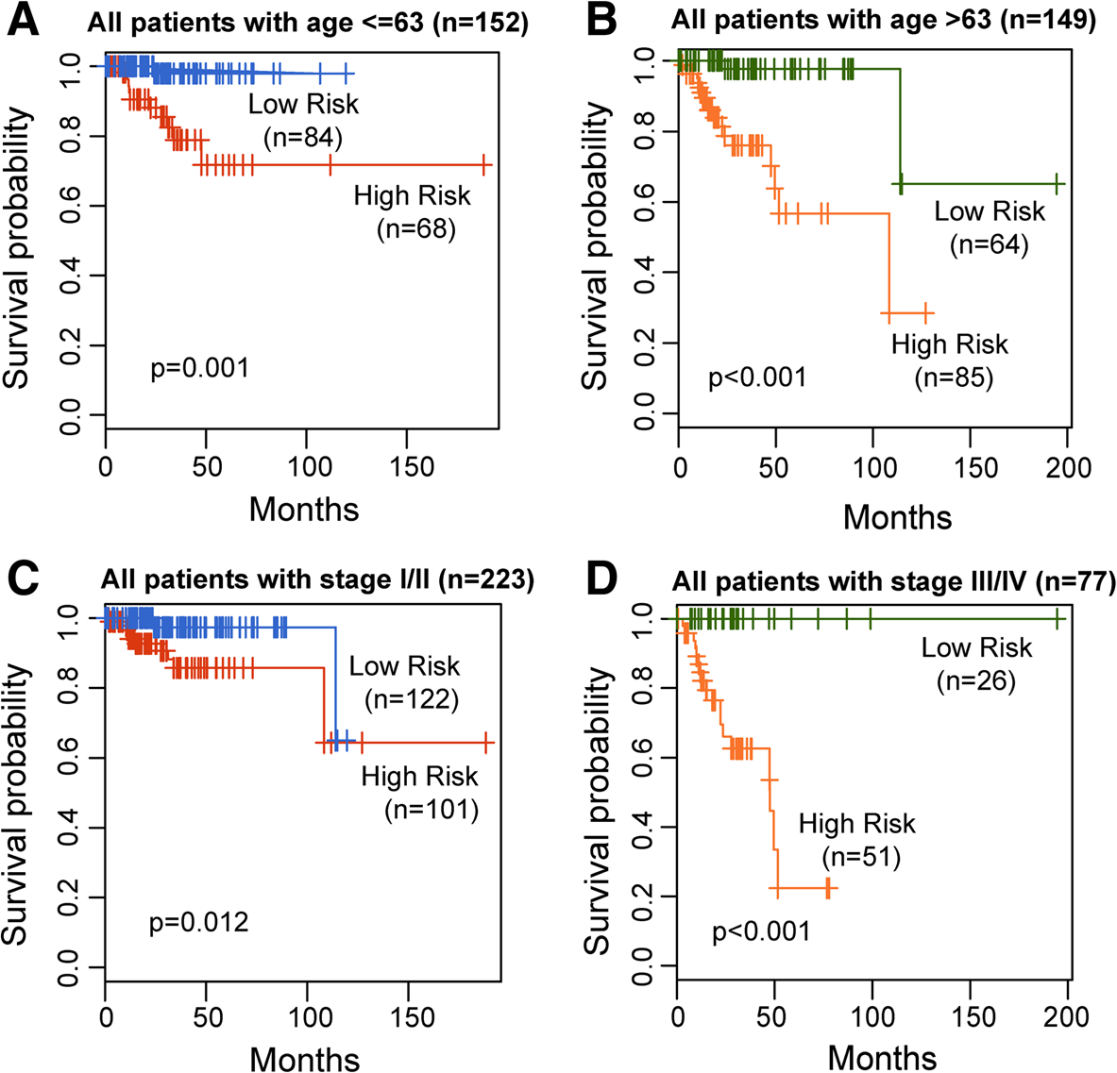
Survival prediction of the lncRNA signature in patients stratified by age and stage. Kaplan-Meier estimates of the overall survival for young patients (**a**) and elder patients (**b**). Kaplan-Meier estimates of the overall survival for patients with early stage (**c**) and with late stage (**d**)

Additionally, we found that age (HR = 1.064, 95% CI = 1.02–1.11, *p* = 0.006) and stage (HR = 3.948, 95% CI = 1.76–8.86, *p* = 0.001) were both significantly prognostic factors associated with survival for all UCEC patients (Table 3). The stratification analysis was performed to ascertain that lncRNA signature was independent of age and stage. The 301 UCEC patients were assigned into a young set (age < =63, *n* = 152) and an old set (age > 63, *n* = 149). For the young set, the lncRNA risk score could further divide patients into a better survival subgroup (*n* = 68) or poorer survival subgroup (*n* = 84) (*p* = 0.001, log-rank test) (Fig. 3a). Patients in the old set exhibit the same trend (Fig. 3b). For elder patients, the LFES also assigned the patients into two subgroups with significantly different survival (*p* < 0.001, log-rank test) (Fig. 3b). The analysis demonstrated that the LFES was free from age. To evaluate whether the LFES may predict the survival of patients within each stage stratum, stratified analysis based on stage was carried out. All UCEC patients were divided into an earlier stage stratum (stage I and II patients) or a later stage stratum (stage III and IV patients). The LFES was performed to distinguish high-risk and low-risk patients in each stage stratum. By the KM curves shown in Fig. 3c and d, patients with high-risk scores have significantly shorter survival than those with low-risk scores for earlier stage stratum (*p* = 0.012, log-rank test) and later stage stratum (*p* < 0.001, log-rank test) (Fig. 3c and d). Multivariate and stratification analysis shows that prognostic power of the LFES was independent of other clinicopathological factors for survival prediction in UCEC patients.

**Fig. 4:**
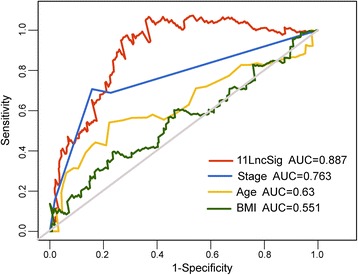
Comparison of sensitivity and specificity for 5-year survival prediction by the lncRNA signature and other clinical factors

We compared the prognostic performance of the LFES with other clinical characteristics used for risk stratification of UCEC patients, including age, stage and BMI. Time-dependent ROC analysis was conducted to compare the sensitivity and specificity of survival prediction. The AUC for each of the prognostic factors was calculated and compared. As shown in Fig. 4, the AUC of LFES was 0.887 that is significantly higher than age (AUC = 0.63), stage (AUC = 0.763) and BMI (AUC = 0.551). These results showed that the LFES had a better prognostic performance than other prognostic factors.

Finally, we compared expression level of 11 lncRNAs in the LFES across four UCEC subtypes (Ultramutated (POLE), Hypermutated (MSI), Low CN (MSS) and High CN (Serous-like)) identified by The Cancer Genome Atlas Research Network based on a combination of somatic nucleotide substitutions, MSI and SCNAs [16]. The results indicated no significant difference in the distribution of expression levels for all 11 prognostic lncRNAs across four UCEC subtypes (Additional file 2), implying that the LFES is not a subtype-specific marker.

### Functional roles of prognostic lncRNAs in the signature in UCEC biology

**Fig. 5:**
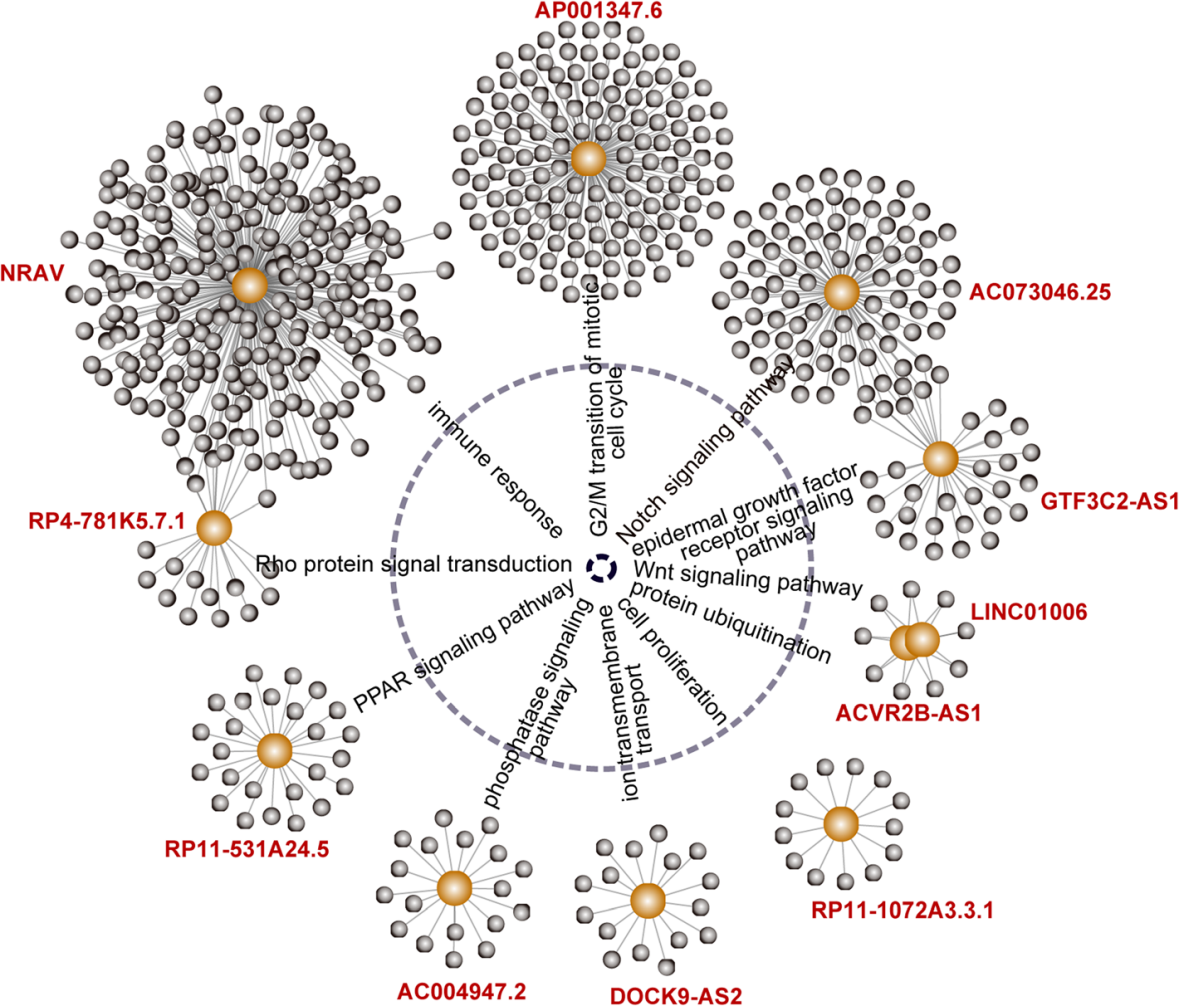
Significantly enriched biological processes and pathways of protein-coding genes correlated with prognostic lncRNAs in the signature

In order to understand functional roles behind the LFES in UCEC biology, we performed in silico analysis for lncRNA function through functional enrichment analysis. An integrated lncRNA-mRNA co-expression network was generated by calculating the Pearson correlation coefficient between expression values of prognostic lncRNAs and those of mRNAs in the entire TCGA patients. Functional enrichment analysis of GO and KEGG was performed for co-expressed mRNAs to infer potential biological processes and pathways of prognostic lncRNAs. We found that these prognostic lncRNAs may be involved in Wnt signaling pathway, Rho protein signal transduction, cell cycle, protein ubiquitination, phosphatase signaling pathway, epidermal growth factor receptor (EGFR) signaling pathway, Notch signaling pathway, immune response, PPAR signaling pathway, ion transmembrane transport and cell proliferation (Fig. 5). It suggested that lncRNAs in the LFES played important roles in UCEC biology.

## Discussion

With the application of molecular profiling, mRNA- or miRNA-focus molecular markers were identified to improve the understanding of the molecular heterogeneity of UCEC and facilitate individualized treatment [19–21]. Recently, altered lncRNA expression has been shown to play critical roles in the development and progression of cancer like miRNAs and protein-coding genes [8, 9, 11, 22–24]. Emerging evidence indicates that lncRNAs are expressed in a more tissue- and cell type-specific manner than protein-coding genes, thus making them attractive as prognostic/predictive biomarkers [11, 25]. During past few years, several lncRNA signatures have been developed to predict the survival of patients with some cancers [25–31]. Although several studies have identified some lncRNAs exhibiting dysregulated expression pattern in UCEC [13–15], these studies were focused on identifying differentially expressed lncRNAs. The prognostic value of lncRNAs for UCEC patients has not been systematically investigated yet.

In our study, we reported a first examination of lncRNA expression profiles at a genome-wide level in a large cohort of patients with UCEC and identified 19 lncRNAs that are significantly associated with overall survival of UCEC patients. A linear combination of 11 independent prognostic lncRNAs (*RP11-1072A3.3.1*, *ACVR2B-AS1*, *RP4-781 K5.7.1*, *AC073046.25*, *AP001347.6*, *DOCK9-AS2*, *NRAV*, *GTF3C2-AS1*, *LINC01006*, *RP11-531A24.5* and *AC004947.2*) was defined as a novel lncRNA-focus expression signature (LFES) to predict survival for UCEC patients. The risk score calculated from the expression of 11 lncRNAs in this signature reveals superior ability to separate patients into high-risk and low-risk groups with significantly different overall survival in both discovery cohort and validation cohort. Furthermore, the LFES is independent of other clinical factors including age, stage, grade and histology and demonstrated better prognostic performance than other clinical characteristics used for risk stratification of UCEC patients. These results indicate that the LFES may be a potential independent predictor to aid in patient-tailored treatment in the future clinical trials.

Although there is a rapid increase in the mapping of lncRNA loci, the elucidation of the biological role of novel lncRNAs is still in his infancy. From our literature review, we found that only one prognostic lncRNAs in the LFES, *NRAV*, has been found to express in numerous human tissues and identified as cancer-related lncRNA in bladder urothelial carcinoma, kidney chromophobe and kidney renal papillary cell carcinoma [32]. A previous study of *NRAV* showed that NRAV was dramatically down-regulated during infection with several viruses and was indicated as a critical regulator of innate immunity [33]. Bioinformatics analysis has been recognized as a commonly used and effective way for elucidating lncRNA function during recent years [34]. Therefore, we performed in silico analysis to infer potential biological roles of prognostic lncRNAs in the LFES by correlating a common expression pattern between lncRNAs and protein-coding genes in all UCEC patients. Functional enrichment analysis for protein-coding genes correlated with a given lncRNA suggested that prognostics lncRNAs in the LFES may be implicated in some key cancer pathways. For example, Wnt signaling pathway, important signaling pathways in the carcinogenesis and embryogenesis, has been implicated in endometrial carcinogenesis [35]. Previous studies have demonstrated a significant correlation of EGFR overexpression with advanced stage and poor prognosis, suggesting that abnormal activation of EGFR signaling pathway contributes to tumorigenesis and metastasis of UCEC [36]. Notch signaling pathway is an evolutionally conserved developmental pathway involved in the regulation of cellular proliferation, differentiation and apoptosis. Jonusiene et al. demonstrated that expression of core elements of the Notch signaling pathway (*NOTCH1*, *NOTCH2*, *NOTCH3* and *NOTCH4*) was down-regulated in UCEC compared to adjacent nontumor endometrial tissue, implying the tumor suppressor roles of Notch signaling pathway in UCEC [37]. In addition, two studies in vivo showed altered expression of PPAR signaling pathway which modulates proliferation and angiogenesis in UCEC [38, 39].

## Conclusions

In conclusion, we identified a novel lncRNA-focus expression signature consisting of 11 prognostic lncRNAs through genome-wide integrated analysis of lncRNA expression profiles and clinical data. The identified 11-lncRNA signatures could be used to robustly predict survival of patients with UCEC. They represent an independent and superior prognostic value compared with the clinical covariates, as shown by multivariate, stratification and ROC analysis. Functional analysis has linked the expression of prognostic lncRNAs to well-known tumor suppressor or oncogenic pathways in endometrial carcinogenesis. With further prospective studies, the lncRNA-focus expression signature provides novel insights into the understanding of the molecular heterogeneity of UCEC and can be valuable biomarkers to improve risk stratification for aiding in patient-tailored selection.

## Additional files

Additional file 1: lncRNAs significantly associated with overall survival in univariate Cox regression analyses. (DOC 38 kb)

Additional file 2: Expression map of the 11 prognostic lncRNAs across four UCEC subtypes. Kruskal-Wallis test was used to compare expression levels for each lncRNAs across four UCEC subtypes. (DOC 765 kb)

## Abbreviations

CI: Confidence intervals
EGFR: Epidermal growth factor receptor
GO: Gene Ontology
HR: Hazard ratios
KEGG: Kyoto Encyclopedia of Genes and Genomes
LFES: lncRNA-focus expression signature
LncRNAs: Long non-coding RNAs
NcRNAs: Non-coding RNAs
ROC: Receiver operating characteristic
TCGA: The Cancer Genome Atlas
UCEC: Endometrial cancer
