# Supplementary material for: A novel lncRNA-focus expression signature for survival prediction in endometrial carcinoma
Source: BMC Cancer. 2018 Jan 5;18:39. doi: 10.1186/s12885-017-3983-0 (PMC5756389; doi:10.1186/s12885-017-3983-0)
Supplement: Supplementary file 1 — lncRNAs significantly associated with overall survival in univariate Cox regression analyses. (DOC 38 kb) [file 12885_2017_3983_MOESM1_ESM.doc]

Additional file 1. lncRNAs significantly associated with overall survival in univariate Cox regression analyses

| **Ensembl id** | **P Value** |
| --- | --- |
| ENSG00000226445 | 0.000 |
| ENSG00000260684 | <0.001 |
| ENSG00000237499 | 0.001 |
| ENSG00000247629 | 0.001 |
| ENSG00000229589 | 0.001 |
| ENSG00000224037 | 0.002 |
| ENSG00000225075 | 0.002 |
| ENSG00000235499 | 0.002 |
| ENSG00000224905 | 0.002 |
| ENSG00000260992 | 0.003 |
| ENSG00000234996 | 0.003 |
| ENSG00000248008 | 0.005 |
| ENSG00000234945 | 0.005 |
| ENSG00000182648 | 0.006 |
| ENSG00000260267 | 0.007 |
| ENSG00000243107 | 0.008 |
| ENSG00000253636 | 0.009 |
| ENSG00000230479 | 0.010 |
| ENSG00000233760 | 0.010 |
