# Supplementary material for: A novel lncRNA-focus expression signature for survival prediction in endometrial carcinoma
Source: BMC Cancer. 2018 Jan 5;18:39. doi: 10.1186/s12885-017-3983-0 (PMC5756389; doi:10.1186/s12885-017-3983-0)
Supplement: Supplementary file 2 — Expression map of the 11 prognostic lncRNAs across four UCEC subtypes. Kruskal-Wallis test was used to compare expression levels for each lncRNAs across four UCEC subtypes. (DOC 765 kb) [file 12885_2017_3983_MOESM2_ESM.doc]

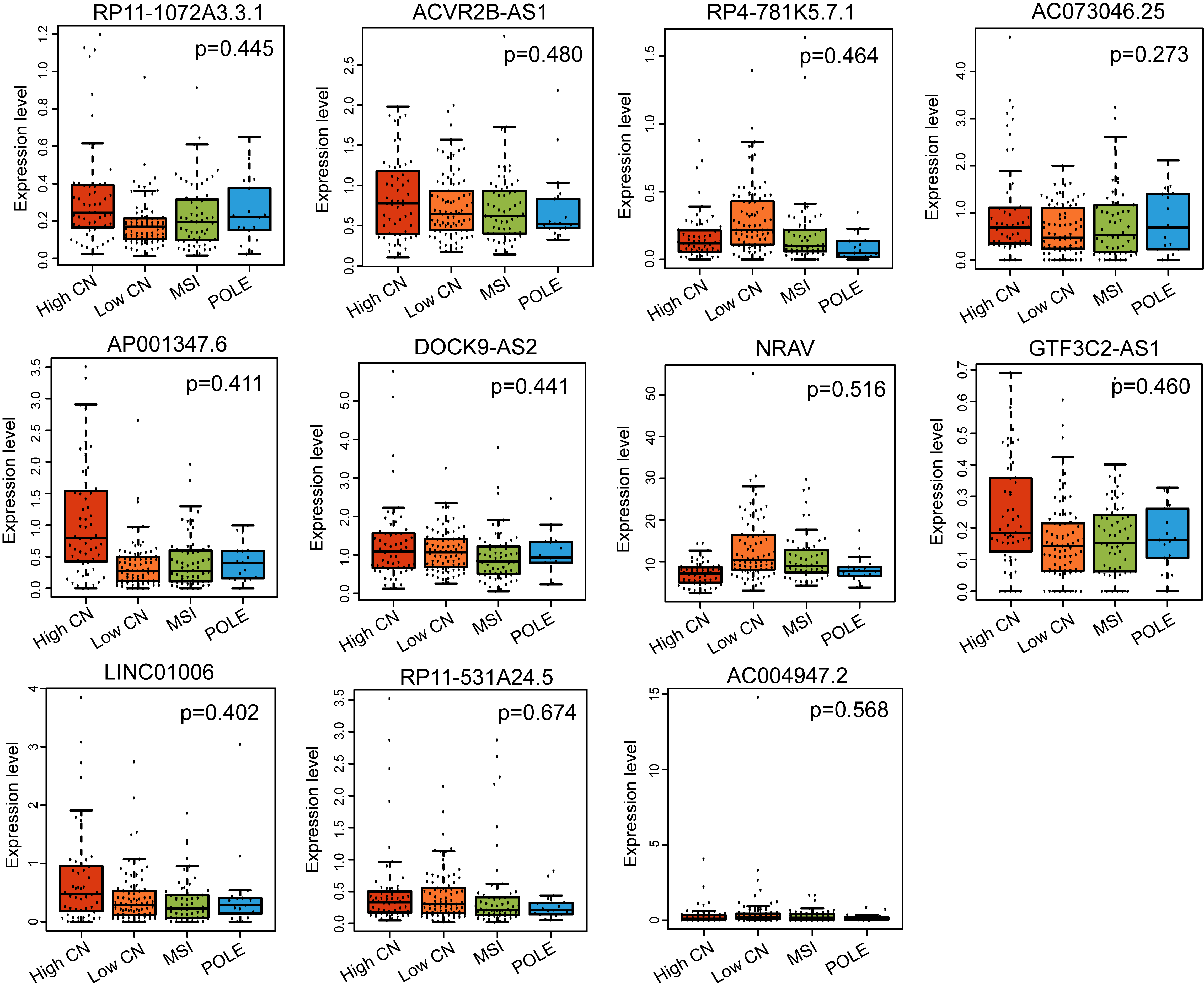


**Additional file 2:** Expression map of the 11 prognostic lncRNAs across four UCEC subtypes. Kruskal-Wallis test was used to compare the expression levels for each lncRNAs across four UCEC subtypes.
